# Supplementary material for: Visualizing functional network connectivity differences using an explainable machine-learning method
Source: Physiol Meas. Author manuscript; Available in PMC 2026 May 18. (PMC13182187; doi:10.1088/1361-6579/adce52)
Supplement: Sup_Info [file NIHMS2169822-supplement-Sup_Info.pdf]

Supplementary tables

Table 1: UK Biobank Dataset Summary

		Middle Aged adults (MA)	Old aged adults (OA)
Site 1 (Site == 11025)	Number	3044	2786
	Age	57.44 ± 4.47	70.04 ± 3.63
	Gender(M/F)	1397/1647	1535/1251
Site 2 (Site == 11026)	Number	517	672
	Age	58.29 ± 4.08	71.23 ± 3.89
	Gender(M/F)	238/279	338/334
Site 3 (Site == 11027)	Number	1136	1239
	Age	58.17 ± 4.04	70.78 ± 3.94
	Gender(M/F)	492/644	611/628

Table 2: FBIRN Dataset Summary

		SZ	HC	P-value
FBIRN Site 1	**Number**	21	28	NA
	**Age**	30.04±8.60	34.78±9.40	0.1
	**Sex(M/F)**	17/4	21/7	0.99
	**PANSS (positive)**	15.72±5.57	NA	NA
	**PANSS(negative)**	14.11±3.14	NA	NA
FBIRN Site 2	**Number**	12	10	NA
	**Age**	44.91±11.34	38.10±9.39	0.14
	**Sex(M/F)**	12/0	7/3	0.62
	**PANSS (positive)**	16.90±6.70	NA	NA
	**PANSS negative)**	17.20±7.39	NA	NA
FBIRN Site 3	**Number**	24	27	NA
	**Age**	44.41±11.90	42.48±12.56	0.57
	**Sex(M/F)**	19/5	21/6	0.99
	**PANSS (positive)**	16.95±4.43	NA	NA
	**PANSS negative)**	16.87±5.91	NA	NA
FBIRN Site 4	**Number**	26	26	NA
	**Age**	36.88±12.82	35.23±10.56	0.61
	**Sex(M/F)**	21/5	20/6	0.99
	**PANSS (positive)**	14.29±3.77	NA	NA
	**PANSS (negative)**	13.41±4.64	NA	NA
FBIRN Site 5	**Number**	14	15	NA
	**Age**	36.64±10.27	37.53±9.76	0.81
	**Sex(M/F)**	9/5	10/5	0.99
	**PANSS (positive)**	13.14±4.46	NA	NA
	**PANSS (negative)**	15±6.28	NA	NA
FBIRN Site 6	**Number**	29	27	NA
	**Age**	36.27±11.08	34.51±10.85	0.54
	**Sex(M/F)**	17/12	16/9	0.99
	**PANSS (positive)**	14.34±4.67	NA	NA
	**PANSS (negative)**	11.93±4.06	NA	NA
FBIRN Site 7	**Number**	25	27	NA
	**Age**	39.56±11.80	37.56±10.75	0.52
	**Sex(M/F)**	20/5	20/7	0.99
	**PANSS (positive)**	16.12±5.41	NA	NA
	**PANSS (negative)**	14.04±6.82	NA	NA
Total	**Number**	151	160	NA
	**Age**	38.06±11.30	37.04±10.68	0.41
	**Sex(M/F)**	115/36	115/45	0.99
	**PANSS (positive)**	15.32±4.92	NA	NA
	**PANSS (negative)**	14.32±5.42	NA	NA
